# Three-Dimensional Geometry Correction of Scratch Grooves via X-Ray Computed Tomography for Fracture Characterization in Cementitious Materials

**DOI:** 10.3390/ma19153192

**Published:** 2026-07-27

**Authors:** Jiahan Liu, Xiayu Zhou, Yu Peng

**Affiliations:** 1 College of Civil Engineering, Shanghai Normal University, Shanghai 201418, China; 1000567242@smail.shnu.edu.cn; 2 College of Civil Engineering and Architecture, Zhejiang University, Hangzhou 310058, China; zjupengyu@zju.edu.cn

**Keywords:** scratch test, X-ray computed tomography, geometric correction, fracture toughness, cementitious materials

## Abstract

Scratch tests have emerged as a promising technique for evaluating the fracture behavior of cementitious materials. However, conventional fracture toughness (*K*_c_) calculations rely on empirical groove geometry models, which may not accurately represent the actual scratch morphology. In this study, X-ray computed tomography (X-CT) and probe profilometry were employed to characterize the three-dimensional morphology of scratch grooves in white cement paste. Scratch tests were conducted under a linearly increasing load up to 30 N. Groove boundaries were identified through grayscale statistical analysis of X-CT images, and the obtained morphological parameters were incorporated into a linear elastic fracture mechanics framework to evaluate fracture toughness. The results showed that the indentation depth increased with the applied load, whereas the lateral force exhibited a nonlinear power-law relationship with the normalized penetration depth. X-CT measurements yield an average elastic recovery rate of 26.05% relative to instantaneous sensor depths, and 15–20% larger groove widths than empirical predictions due to edge spalling and debris accumulation, with discrepancies that amplify with increasing load. The X-CT-corrected *K*_c_ averages 0.21 MPa⋅m^1/2^, about 30% lower than values derived from empirical geometric fitting. Good agreement between X-CT and probe profilometer measurements validated the reliability of the proposed approach. The main contribution is the correction of the *K*_c_ calculation using actual geometry. The results demonstrate that X-CT is an effective and non-destructive method for accurately characterizing fracture toughness evaluation of cementitious materials.

## 1. Introduction

Fracture toughness is a critical intrinsic property governing the durability and service life of cement-based materials (CBMs), particularly in aggressive environments such as marine infrastructure and architectural conservation [1]. Conventional macro-scale methods for characterization, e.g., three-point bending, wedge splitting tests, require bulky specimens with characteristic dimensions exceeding 40 mm to satisfy the small-scale yielding criterion of linear elastic fracture mechanics (LEFM) [2,3]. This fundamental limitation precludes their application to small-volume samples, including drill cores from existing structures, thin repair layers, and heritage building materials, where localized property assessment is urgently needed.

The microscratch test has emerged as a promising, requiring only millimeter-scale specimens to probe localized fracture behavior. This technique has been extensively applied across various scientific and engineering fields to evaluate the hardness and wear of polymeric materials [4,5], deformation behavior of metals [6,7], interface strength of composites [8], mechanical mechanism of inorganic materials [9], adhesion/damage mechanism of thin films/coatings [10,11,12,13,14,15], and strength of rocks/cement-based materials (CBMs) [16,17,18]. Research has indicated that scratch tests can not only assess the strength characteristics of materials but also their fracture behavior. The foundational LEFM framework for microscratch-based evaluation was established by Akono and Ulm, who derived a closed-form solution relating lateral force to fracture toughness via assumed idealized groove geometries [19,20]. This calculation method were applied to study the fracture properties of various materials, including minerals, metals, and polymers [21,22]. This linear elastic calculation model can be further extended to study the fracture properties of heterogeneous materials, such as CBMs. Akono et al. determined the stress intensity factor (SIF) of hardened cement paste (HCP) through a scratch test [20]. Liu et al. used nanoscratches to characterize the interfacial microstructure between basalt fiber and polymer-modified cement and Portland cement, which confirmed that the interface has high fracture toughness [23]. Subsequent studies extended this framework to phase-resolved quantification. Yao et al. integrated microscratch testing with scanning electron microscopy (SEM) to establish quantitative correlations between bulk brittleness and C-S-H phase properties [18]. Němeček et al. advanced phase-resolved characterization via microscratch coupled with acoustic emission, reporting values of 0.54–1.24 MPa⋅m^1/2^ across cement paste constituents [24]. However, a universal bottleneck persists across all existing microscratch studies: the groove geometry is approximated by empirical formulas that ignore three-dimensional (3D) damage features ubiquitous in cementitious materials. Some physical phenomenons (edge spalling, debris accumulation, and subsurface microcracking) will affect the geometric parameters (width, depth, cross-sectional area), which are inherent to the fracture process zone (FPZ) of quasi-brittle cementitious materials [25]. For the sphero-conical (Rockwell) indenter widely used for cementitious composites, existing calibration methods based on dimensionless fitting of known materials fail to account for elastic recovery and creep effects, leading to non-negligible overestimation [26]. To date, no prior work has quantified the magnitude of this geometric bias or developed a correction protocol leveraging 3D imaging. Consequently, refining the approach for determining the scratch groove morphology can improve the calculation of the mechanical properties of composites.

X-ray computed tomography (X-CT) enables non-invasive, three-dimensional (3D) visualization of internal structures by reconstructing volumetric data from a series of two-dimensional (2D) X-ray projection images [27,28,29,30]. It is widely applied in fields such as medical diagnosis and non-destructive testing of materials. Owing to the capacity of X-rays to penetrate dense, non-transparent materials, X-CT is especially effective for examining cementitious materials properties, including microstructures, porous networks, fiber distributions, crack development, transport properties and workability [29,31,32,33,34]. Leite et al. studied the microstructure of recycled concrete (RC) with SEM using synchrotron microtomography with an advanced light source [29]. This study verified the influence of RC aggregates and their water absorption compensation on the RC microstructure. Kucharska and Dybel employed X-CT to show that self-compacting concrete (SCC)–rebar interface degradation under top bars stems from proximity to the cast surface rather than element size, challenging standard assumptions linking bond quality to concrete depth [33]. Recent advances have successfully coupled X-CT with mechanical simulations. Zhang et al. established X-CT-image-based mesoscopic finite element models to simulate concrete fracture under dynamic splitting, revealing the shift from interfacial to aggregate failure [32]. Zhang et al. extracted 30 virtual specimens from X-CT 3D images of cement paste and computed stochastic tensile strengths via lattice modeling, bridging microstructure to macro-scale fracture behavior [35]. Zeng et al. employed X-CT to trace the entrapped mercury in two HCPs after mercury intrusion porosimetry tests to reassess the method for pore structure characterization [31]. Nevertheless, these X-CT-based efforts have focused exclusively on macro-scale fracture processes or numerical model construction. The application of X-CT to microscratch groove reconstruction and subsequent LEFM parameter correction remains entirely unexplored.

Despite the work on microscratch testing and X-CT characterization, a critical scientific gap persists: the absence of a validated framework linking X-CT-resolved 3D scratch groove morphology to LEFM-based calculation, and the lack of quantitative understanding of how geometric biases propagate into errors for CBMs. Hence, the objectives of this study are to: (1) quantify the discrepancies between empirical geometric assumptions and X-CT-resolved 3D groove morphologies for CBMs; (2) establish an X-CT-corrected LEFM framework for microscratch evaluation; (3) validate the corrected values against stylus profilometer measurements and conventional fracture mechanics benchmarks; and (4) elucidate the micro-mechanisms of scratch-induced fracture via integrated X-CT and SEM analysis. This work represents the systematic attempt to bridge microscratch testing with X-CT-based 3D characterization for cementitious materials, providing a reliable pathway for accurate assessment of small-volume samples. The findings of this study provide promising advances in testing fractures by scratch tests.

## 2. Materials and Methods

### 2.1. Materials and Specimen Preparation

Cement paste specimens were fabricated using P.W 52.5 white cement (WCP, Alborg, Anqing, China) as the binding material, in accordance with the Chinese national standard GB/T 2015–2005 [36]. Detailed information regarding the chemical composition and physical properties of the WCP is presented in Table 1 and Table 2, respectively. A w/c mass ratio of 0.4 was adopted, employing ordinary tap water—no rheology-modifying admixtures were introduced. After thorough homogenization, the fresh paste was cast into molds measuring 40 mm × 40 mm × 160 mm. Curing was carried out under controlled environmental conditions (a constant temperature of 20 °C and relative humidity exceeding 95%), which were sustained for 28 days. To halt ongoing hydration, the hardened samples were immersed in anhydrous ethanol for one week—a process that effectively displaces residual pore water. Finally, the specimens were subjected to thermal drying at temperatures above 50 °C to remove moisture retained within capillary pores or adsorbed onto internal pore surfaces [37].

### 2.2. Scratch Test

Scratch tests were performed using a micron-scale scratch tester (MST, Anton Paar, Baden, Switzerland) equipped with a Rockwell-type diamond indenter. This indenter had a spherical tip radius of 100 μm and a conical half-angle of 60°. A linearly increasing load (*F*_V_ ∝ t) was applied, reaching a peak force of 30 N. Based on the author’s previous research on cement-based materials and studies by Akono and Ulm [38,39], the peak force of 30 N is sufficiently large to ensure stable crack propagation while avoiding excessive specimen damage, and the generated groove can also satisfy the resolution requirements of the following X-CT image analysis. For each sample, three parallel scratches—each 3 mm in length and traversed at a constant velocity of 6 mm/min—were made. The scans conducted before and after the experiment were both performed under a load of 0.03 N. The scratch speed in this scratch test is categorized as quasi-static loading, ensuring that the energy dissipation is dominated by fracture processes. According to ASTM C1624 [40] and the established method in the microscratch literature for HCP, three scratches can ensure statistical representativeness. Throughout the test, high-precision transducers continuously tracked both the transverse force (*F*_T_) and the instantaneous penetration depth (*d*). Data acquisition occurred at a frequency of 30 Hz, yielding measurement points spaced at precisely every 3 μm along each scratch track. To ensure environmental stability and minimize external interference, all experiments were carried out inside a sound-isolated chamber maintained at a steady temperature.

### 2.3. X-Ray Computed Tomography

Owing to the brittle nature of CBMs, noticeable peeling occurred at the groove boundary during the scratch test. This phenomenon makes accurate determination of the width of the scratch groove using backscattered electron (BSE) image analysis difficult, and only an approximate range of the groove width can be identified. To determine the morphological parameters of the grooves, 3D reconstruction of the scratch region was performed using X-CT image analysis.

The specimens were scanned by a Nikon XTH 255/320 LC (Nikkon, Tokyo, Japan) X-CT scanner equipped with a microfocusing composite metal target. A high-sensitivity DRZplus scintillation detector, positioned behind the specimen, was employed to quantify the photon beam intensity, offering a spatial resolution of 2000 (h) × 2000 (v). Before the scanning process was initiated, complete preparatory procedures, including specimen installation and positioning, system preheating, preliminary configuration of scanning parameters, energy calibration, determination of exposure time, and detector calibration, were essential. High-performance computers were used along with the corresponding software and detectors to store data to reconstruct and display the image data. To acquire high-quality image data, a stable X-ray source and detector, appropriate voltage and energy settings, and suitable exposure and total scanning durations must be employed [41]. The distance between the turntable and the X-ray source was set to the minimum possible value, at which the image achieved the smallest voxel size of 5 μm. Appropriate adjustments should be made according to the dimensions of the test specimen to ensure optimal imaging results.

For the WCP specimens in this study, a voltage of 200 kV and a tube current of 100 μA were used. The 2D projection image was acquired through scanning at angular intervals of 0.12°, and the exposure time for each projection was 500 ms. In total, 3000 projections were generated to complete 360 degrees of scannings. The acquired 2D projection images were imported into VGSTUDIO MAX software (version 3.1, Volume Graphics, Inc., Charlotte, NC, USA) for image reconstruction using 8-bit grayscale values via the Feldkamp–Davis–Kress (FDK) cone-beam algorithm. This method has been verified in Zeng et al. [42] and Wang et al. [43] for cement paste systems. To mitigate artifacts inherent to polychromatic X-ray sources and detector inhomogeneities, two preprocessing filters were applied prior to/during reconstruction: beam hardening correction and ring artifact reduction. After the complete 3D model was obtained, the scratch area was sectioned along the scratch direction into 300 slices for the HCP specimens. Segmentation was performed on the 3D volume to extract the scratch groove from the cement paste matrix, using a global thresholding approach validated by the grayscale histogram. All segmentation-derived geometries represent lower-bound estimates of the true scratch groove morphology, as X-CT reconstruction is subject to inherent uncertainties. The 5 μm isotropic voxel size, optimized for 5 mm thick specimens, introduces a maximum 2.5% measurement bias for macro-scale grooves (200–500 μm wide) due to voxel averaging. Features ≥10 μm (2 voxels) are reliably resolved, but microcracks narrower than 5 μm are blurred and cannot be quantified. Beam hardening correction and ring artifact reduction suppressed noise to <1%, and the filtered data showed <4% deviation from profilometer measurements, confirming minimal noise impact. The FDK reconstruction algorithm, which is the standard for cement-based X-CT, incurs a 1–2% error for homogeneous specimens [44]. The results indicate that X-CT can accurately capture the 3D morphological characteristics of the scratch area.

### 2.4. Probe Profilometer

To verify the accuracy of using X-CT technology in characterizing the morphology of surface grooves, a probe profilometer was used to measure the profiles on the surface of the specimens. The results were compared with those obtained from X-CT image analysis. Probe profilometry is widely employed for measuring film thickness, stress, surface roughness, and surface topography [45,46]. The specimens were tested by a probe profilometer (KLA-Tencor D-100, Tencor, San Jose, CA, USA) with an LIS 3 transducer. The probe diameter is 2 μm, and the vertical accuracy is 0.1 nm. Because the probe profilometer cannot perform equal-distance sectioning of the scratch area, ten sections were randomly selected along the entire length of each scratch groove. The displacement data acquired through the probe profilometer were converted into morphology data representing the scratch groove.

## 3. Morphological and Fracture Property Characterization

### 3.1. Scratch Profiles and Characteristic Outcomes

**Figure 1:**
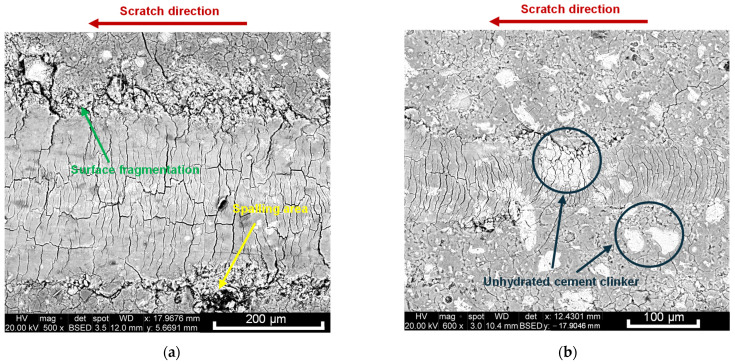
BSE image of the scratched region on the surface of the WCP specimen for (**a**) the rear and (**b**) the front areas of the scratch.

A BSE image depicting the scratched area on the surface of the WCP specimen is presented in Figure 1. The WCP consisted of uniformly dispersed unhydrated cement particles embedded in a continuous hydration product matrix. Scratch grooves formed on the specimen surface, with both the depth and width gradually increasing as the load increased [38,47]. The material accumulated along both sides of the scratch track. Since the load applied during the formal scratch test was significantly greater than that applied before scratching, the depth before scratching was considerably less than the formal scratch depth. Consequently, the instantaneous indentation depth was used to determine vertical displacement.

**Figure 2:**
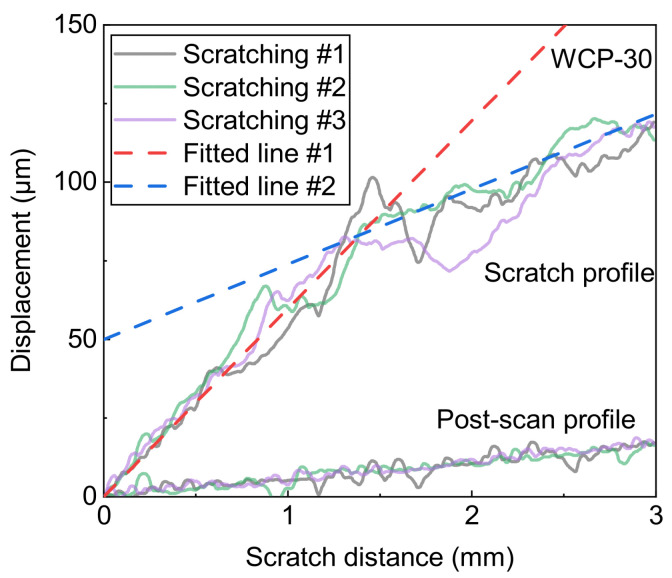
Profile of the WCP specimen from the formal and post-scan scratch tests along with the curves fitted to the displacement versus scratch distance.

Figure 2 presents the results of the scratch test and the post-scan vertical displacement of the WCP specimen along the scratch direction. A distinct linear relationship was observed between the indentation depth and vertical load, particularly at lower loading conditions (up to approximately 15 N). As the load increased, the relationship remained linear but showed a steeper slope, indicating a fundamental change in mechanical response. The quantitative fitting parameters for the displacement–scratch-distance curve and the statistical analysis are summarized in Table 3. This transition is likely linked to an evolving dominance among competing deformation mechanisms—namely elastic deformation, plastic deformation, and fracture processes. Consequently, the relative contribution of each mechanism to the overall material response changed progressively throughout the test. The slopes of the linear fitting equations derived from WCP exhibit consistent trends: in the initial loading stage, slope values ranged from 40 to 65; in the later stage, they decreased to 20–35. Furthermore, under identical applied loads, WCP has demonstrated an indentation depth distribution comparable to that of ordinary Portland cement (OPC) during scratch tests, which was approximately distributed around 80–150 μm [38].

**Figure 3:**
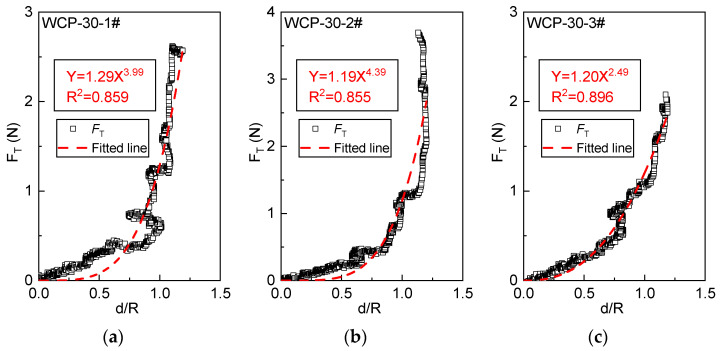
Nonlinear fitting relationship between *F*_T_ and *d*/*R* during the scratch test for the (**a**) WCP-30-1#, (**b**) WCP-30-2# and (**c**) WCP-30-3# scratch.

To gain deeper insights into how lateral load correlates with indentation depth during scratch testing of WCP, a nonlinear power-law relationship was formulated between *F*_T_ and *d*/*R*. As illustrated in Figure 3, the curve-fitting analysis confirmed this nonlinearity. Specifically, *F*_T_ scales with (*d*/*R*) according to a power function:

(1) $$Y=aX^{b}$$

where *a* and *b* are the fitting parameters. The least square method was used to fit the resulting curve to obtain the estimated power function equation, which demonstrated that the value of *b* fell in the range of 2.49–4.39. Akono et al. demonstrated that the geometry of the Rockwell indenter can exert a pronounced influence on scratch test results across multiple length scales—ranging from the micro- to macro-scale—and across diverse materials, such as ceramics, glasses, metals, and polymers [48]. For metal and cementitious materials, the relatively deep indentation leads to a *d*/*R* ratio greater than 0.15, implying that the active contact region of the indenter during scratching behaves more like a cone, with the shape factor *b* exceeding 1.5. For anisotropic materials, the value of *b* can not only exceed 1.5 but also display substantial variability, attributable to inherent heterogeneity and directional dependence in mechanical behavior, as the WCP in this study shows. Compared with OPC, parameter *b* is significantly larger, indicating that the composition and properties of WCP are different from those of OPC [38]. Since the value of parameter *b* is related to the uniformity of the material, the fitting result for WCP suggests that its non-uniformity is significantly higher than that of OPC. This might be because WCP contains not only the main components but also gypsum and other components. The fitting parameter *a* exhibits a direct relationship with the material’s fracture behavior. In particular, an increase in the parameter *a* leads to a corresponding rise in the SIF [48]. The parameter *a* of WCP ranges from 1 to 2, which is markedly lower than that of OPC. This suggests that the fracture property of WCP is substantially weaker than that of OPC.

### 3.2. Fracture Toughness Characterization

Based on the LEFM theory, fracture toughness (*K*_c_) of materials can be calculated using the scratch test results [20]:

(2) $$K_{c}=\frac{F_{T}}{\sqrt{2pA_{\mathrm{LB}}}}$$

**Figure 4:**
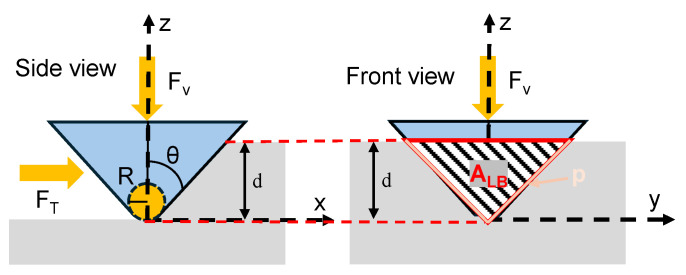
Scratch test performed at a controlled vertical loading rate. Here, the *x*-axis represents the transverse scratching direction, the *z*-axis represents the vertical loading direction, *F*_V_ is the vertical load, *F*_T_ is the lateral load, *R* is the indenter tip radius, *θ* is the half-apex angle, *d* is the scratch penetration depth, *p* is the side length of the projected area, and *A*_LB_ is the contact area along the scratch direction.

**Figure 5:**
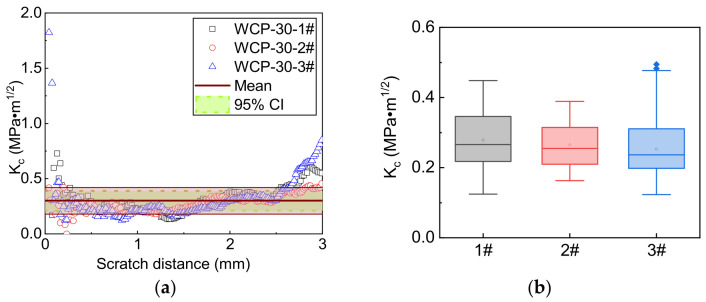
(**a**) Fracture toughness obtained via scratch tests on WCP specimens and (**b**) ANOVA analysis.

The definition of each parameter is presented in Figure 4. Parameters *p* and *A*_LB_ cannot be directly obtained through measurements when calculating the fracture toughness. Studies have shown that, for a conical or spherical probe, a proportional relationship exists between 2*pA*_LB_ and *d*^3^ (2*pA*_LB_ ∝ *d*^3^) [48]. When using the same probe, this relationship remains consistent across different scratched materials. Consequently, if the fracture toughness of a specific material is known and the correlation between 2*pA*_LB_ and *d* is established through a scratch test, the fracture toughness of other materials tested with the same probe can be derived [49]. For the WCP specimens evaluated in this study, the epoxy resin was selected as the reference, which has a known fracture toughness of 0.621 MPa⋅m^1/2^ [38]. *K*_c_ progressively converged to a stable value, as shown in Figure 5a. The values of *K*_c_ and the statistical parameters (e.g., mean, error, SD, and 95%CI) are shown in Table 4. A one-way analysis of variance (ANOVA) was conducted to evaluate the differences in the calculated fracture toughness among the three specimens, as shown in Figure 5b. The results indicated a statistically significant difference between the groups due to the inherent microstructural heterogeneity of cementitious materials. However, the large sample size ensures the robustness of the observed statistical significance. This analysis confirms that the differences in fracture properties between the groups are not attributable to random chance.

According to Figure 1, the uniform dispersion of unhydrated cement particles in the matrix confirms the high microstructural homogeneity of the WCP specimens, providing a micro-scale rationale for the low inter-replicate variability of fracture toughness reported in our statistical analysis. In addition, the scratch groove surfaces exhibited discontinuous, tortuous microcracks, which validate the quasi-brittle nature of scratch-induced fractures in WCP. The fitting width and the tested depth of the transducer during the scratch test were applied to the previous analysis of fracture properties. The results are shown as a red dotted line in the following figures for comparison.

The limitation of the LEFM model lies in its oversimplified assumptions regarding crack propagation, neglecting the variable actual crack paths caused by the contact conditions and material heterogeneity. Moreover, fracture toughness test results demonstrate a size effect. The obtained fracture toughness values are influenced by the geometric dimensions of the test specimen. The values obtained from the scratch model are apparent fracture toughness values based on LEFM theory. In this study, we justify the application of LEFM because the scratch-induced crack length (hundreds of microns) is an order of magnitude larger than the maximum grain size (~10 µm) and the estimated FPZ size (~20–50 µm), thus minimizing the scale effects. It satisfies Bazant’s size effect criterion for LEFM applicability [50]. WCP exhibits a uniform microstructure with homogeneously dispersed unhydrated clinker, which confirms the microscopic homogeneity, justifying LEFM’s homogeneous medium assumption, as shown in Figure 1. While advanced fracture models offer superior capabilities for capturing complex damage, they are incompatible with the objectives of this study: the Extended Finite Element Method (XFEM) relies on predefined crack growth criteria, which are numerically unstable for the branched, curved cracks resolved by X-CT [51]. Nonlinear fracture mechanics (e.g., double-K model) requires distinguishing between crack initiation and stable propagation loads. However, microscratch load-depth curves exhibit no clear initiation inflection point, preventing separation of initiation and unstable toughness values [52].

### 3.3. X-CT Analysis

**Figure 6:**
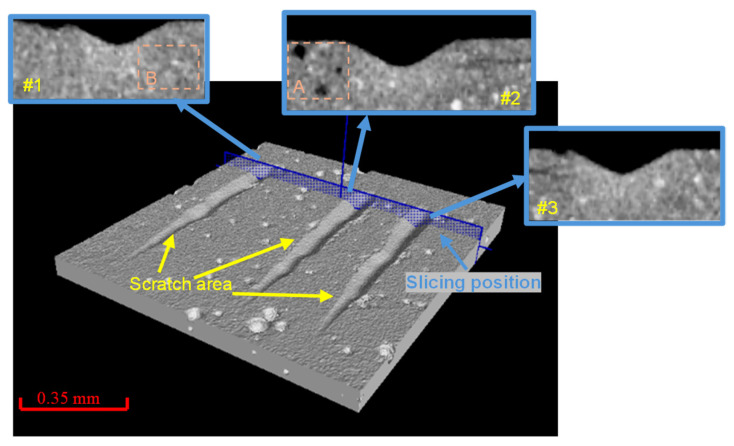
Reconstructed X-CT 3D image and 2D slice image along the scratch direction for the WCP scratch area.

**Figure 7:**
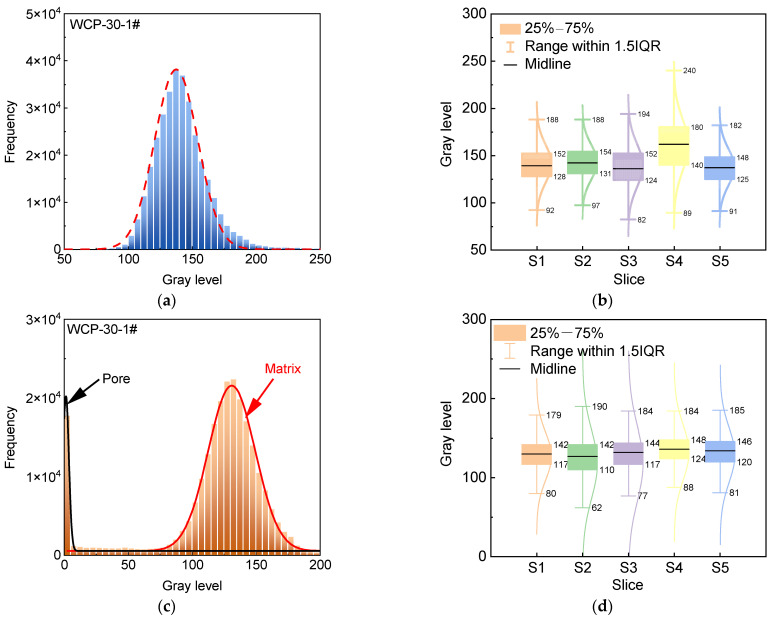
Statistical distribution of gray values of WCP specimen matrix cross-sections (**a**,**b**) containing pores and (**c**,**d**) without obvious pores. The red dashed line is the fitted normal distribution curve. Different colors in (**b**,**d**) means different slices of the specimen.

The reconstructed X-CT 3D image and a 2D slice image along the scratch direction for the scratch area are shown in Figure 6. The slice images were processed using MATLAB (R2021b) to extract the gray value corresponding to each pixel. The gray value range of the scratch groove region can be measured by the statistical distribution of gray levels across the cross-section. CBMs are typically heterogeneous materials and contain internal pores or microcracks. To more accurately define the boundaries of the grooves, matrix regions with minimal porosity and those with obvious porosity from the X-CT images were chosen for analysis. Part A in Figure 6 illustrates the matrix regions with obvious porosity, whereas Part B illustrates those with minimal porosity. The three scratch tests conducted on the WCP were performed with the same acceleration voltage and current, so the distribution range of gray values was consistent. For each scratch, five matrix cross-sections containing pores and five without obvious pores were randomly selected for statistical analysis. The gray value at the scratch boundary can be determined by identifying the minimum gray value. Figure 7 shows the statistical analysis results for one WCP specimen. Due to the distinct X-ray attenuation disparity between the solid cementitious matrix and the pores, the cross-sectional gray-level distribution of the HCP containing pores exhibited two distinct peaks, whereas the matrix region without pores only displayed a single peak. This peak corresponds to one of the peaks shown in Figure 7c, indicating that the two peaks represent the gray-level distributions of the matrix and the pores. The gray-level values of the matrix in the WCP mainly ranged from 70 to 255, whereas those corresponding to the pore phase were primarily between 0 and 30. To eliminate subjective bias, the optimal threshold of 70 was mathematically determined using Otsu’s algorithm, which calculates the threshold that minimizes the intra-class variance while maximizing the inter-class variance of the grayscale histogram [53]. Consequently, the value of 70 was selected as the boundary to analyze the morphology of the grooves in the scratch test for the WCP specimens in this study. Wang et al. have reported that ±5% threshold shift will causes <2% volume change, verifying the threshold robustness for similar cementitious systems [43]. Based on the range of the obtained gray values, the pixel positions corresponding to the groove locations can be identified. When combined with the actual physical length represented by each pixel in the image, the morphology of the scratch groove can be quantitatively analyzed.

**Figure 8:**
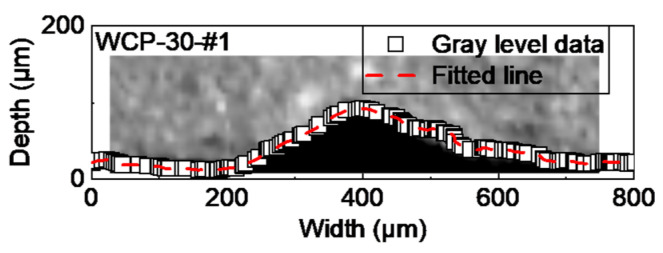
Geometric morphology of the groove with the original grayscale slice.

For each scratch groove on the WCP specimen, nine cross-sections were uniformly selected along the scratch direction. The statistical gray value distribution of the 2D X-CT image slices at these locations was analyzed. The gray value distributions of all cross-sections also exhibited a bimodal pattern, corresponding to the scratch groove and the cement matrix. This suggests that the grayscale information contained in the cross-sectional images can effectively represent the geometric morphology of the scratch groove in CBMs. The morphology of the grooves, including parameters such as depth and width, at the corresponding positions of the HCP specimen is shown in Figure 8. The robustness of this threshold was further validated by overlaying the obtained data curve onto the original grayscale slice. The results indicate that the groove shape is consistent with that of the indenter, exhibiting an approximately conical profile. The edge of the groove formed in the CBMs was noticeably smoother, but the edge exhibited protrusions. These protrusions resulted from the accumulation of debris during the scratching process. This phenomenon suggests that the CBMs have certain elastic properties.

**Figure 9:**
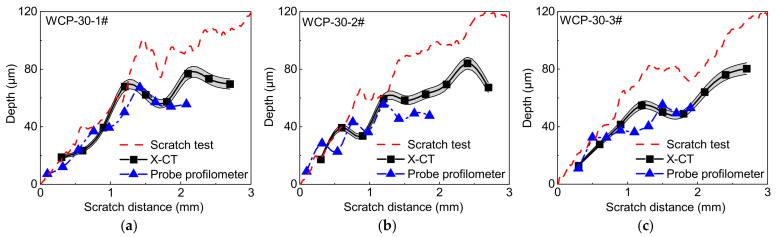
Indentation depth of the (**a**) WCP-30-1#, (**b**) WCP-30-2# and (**c**) WCP-30-3# scratch along the scratch distance measured by transducer during scratch test, X-CT image analysis and probe profilometry.

Based on the X-CT image analysis, morphological parameters such as the indentation depth and width at the corresponding groove positions can be measured. Figure 9 presents the indentation depth of three WCP specimens along the scratch distance measured by the transducer during the scratch test and X-CT image analysis. The depth values gradually increased with increasing load. There was notable discrepancy between the scratch depth measured through X-CT and that obtained from the transducer. The values derived from X-CT were obviously lower. As the applied load increased, the discrepancy between the two measurement methods became more pronounced. This phenomenon is inferred to be attributable to the combined effects of elastic deformation, plastic deformation, and fracture during scratching. The time-dependent behavior of cementitious materials suggests that partial recovery of elastic deformation occurs during the interval between the scratch test and X-CT measurement, which likely contributes to the observed depth reduction. Furthermore, the depth of the scratch increased with increasing applied load, resulting in more obvious deformation recovery. The percentage difference between the depth *d*_XCT_ obtained from the X-CT image analysis and the instantaneous indentation depth *d* measured by the sensor during the scratch test can be used to calculate the elastic recovery rate *R*_g_:

(3) $$R_{g}=\frac{d-d_{\mathrm{XCT}}}{d}\times 100\%$$

**Figure 10:**
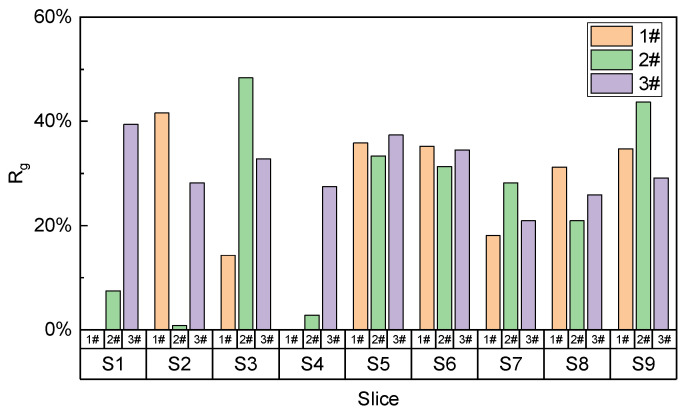
Elastic recovery rates.

The elastic recovery rates computed across nine cross-sectional locations for each specimen are presented in Figure 10. The maximum observed value was 48.32% while the overall mean was 26.05%. The elastic recovery in WCP is decoupled into three synergistic microstructural processes. The first reason is the reversible deformation of C-S-H gel. Two reversible mechanisms operate at the nano-scale: (i) short-range elastic rebound of the C-S-H solid skeleton, governed by van der Waals interactions between calcium silicate sheets, and (ii) reversible migration of interlayer water within the C-S-H gel pores, which is redistributed to accommodate unloading-induced stress release [54]. Another reason may be the partial closure of microcracks. The SEM images in Figure 1 reveal tortuous microcracks propagating along the unhydrated cement particles and the C-S-H matrix. During loading, these cracks open reversibly under the compressive–tensile stress field beneath the indenter; upon unloading, the elastic rebound of the surrounding matrix will drive closure of these microcracks. The third reason may be the stress redistribution within the cementitious matrix. WCP exhibits a uniform microstructure with homogeneously dispersed unhydrated clinker grains and low total porosity. During loading, stress concentrates at pore boundaries and particle–matrix interfaces; upon unloading, the high-modulus unhydrated clinker grains act as elastic constraints, driving stress redistribution from the stiff clinker phase to the compliant C-S-H matrix. This process synchronizes the reversible deformation of individual phases, resulting in macroscopically observable elastic recovery [55]. Regarding the contribution of each component, porosity provides limited space for reversible deformation and C-S-H is the primary contributor to elastic recovery.

**Figure 11:**
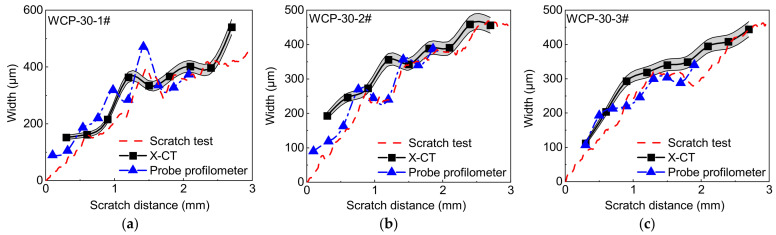
Widths of the scratch grooves on the (**a**) WCP-30-1#, (**b**) WCP-30-2# and (**c**) WCP-30-3# scratch along the scratch distance measured by the transducer, X-CT image analysis and probe profilometry.

The groove width calculated based on the indentation depth obtained from the transducer was compared with the data obtained through X-CT image analysis, as shown in Figure 11. The width gradually increased with increasing applied load, and the width measured via X-CT was greater than that derived from the empirical formula. This discrepancy is inferred to reflect edge spalling and debris accumulation along groove boundaries, as visually observed in X-CT cross-sections. Edge spalling is the dominant mechanism causing the greater width captured by X-CT. During scratching, the compressive stress field ahead of the indenter causes chunks of the cementitious matrix to detach from the groove edges, forming irregular protrusions that are clearly resolved in X-CT cross-sections, as shown in Figure 6, which was verified by probe profilometer analysis in the following section, Section 3.4. The spalled fragments are often displaced laterally, permanently widening the groove. Surface fragmentation also contributes to the greater depths. The brittle nature of WCP leads to the generation of fine debris particles on the groove surface. Some of these fragments are ejected sideways and adhere to the groove shoulders, increasing the apparent width when segmented by X-CT. SEM confirmed the presence of loosely attached debris particles along the groove margins, as shown in Figure 1. Because the scratches produced on the specimen surface under a relatively low load are difficult to observe at the minimum resolution of the X-CT instrument, the groove width measured at the end of scratch initiation has a relatively large error.

**Figure 12:**
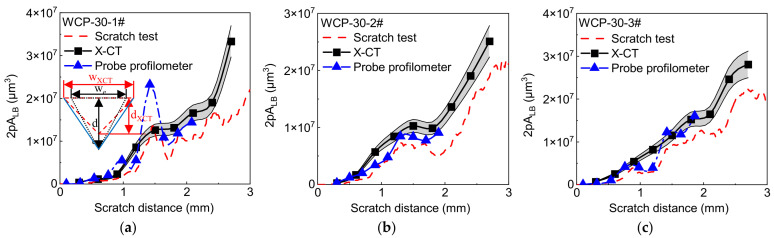
2*pA*_LB_ values of the scratch grooves on the (**a**) WCP-30-1#, (**b**) WCP-30-2# and (**c**) WCP-30-3# scratch along the scratch distance measured by the transducer, X-CT image analysis and probe profilometry.

The results of the shape parameter 2*pA*_LB_ of the scratch groove on the WCP specimen obtained by fitting with the empirical formula were compared with those obtained by X-CT image analysis, as shown in Figure 12. The morphology of the scratch test groove on the WCP obtained from X-CT image analysis differed significantly from the results measured by the transducer and calculated by empirical formulas. Therefore, considering the influence of parameter selection on the accuracy of fracture property calculations is essential. According to the procedure for using the LEFM model with the scratch test, the morphology of the groove was closely associated with the crack propagation region during the scratch process [52,56,57,58]. Energy release occurs during the scratch test. Therefore, when determining fracture toughness, accounting for actual crack propagation during tests is essential. That is, the instantaneous indentation depth (*d*) measured by the transducer and the width obtained from X-CT (*w*_XCT_) were chosen for calculation. A schematic diagram is shown in Figure 12a. The variation trends of the calculated shape parameters with increasing load were consistent. X-CT revealed that at the same groove depth, the groove morphology parameters increased because of the increase in width determined by X-CT. At the start of the test, that is, when the groove area was small, the corrected scratch groove morphology parameters were essentially consistent with the results calculated via the empirical formula. As the load increased, the groove area gradually increased, and the discrepancy between the two sets of results gradually widened. Therefore, accurately characterizing the morphology of grooves formed during scratch tests on CBMs—particularly parameters under high loads—is inferred to be important for precisely determining the material’s fracture properties, as geometric errors propagate directly into the LEFM-based *K*_c_ calculation.

**Figure 13:**
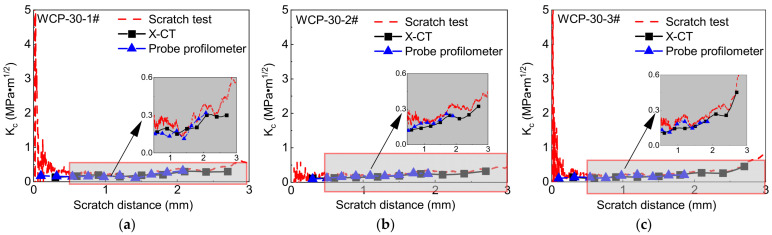
*K*_c_ values of the scratch grooves on the (**a**) WCP-30-1#, (**b**) WCP-30-2# and (**c**) WCP-30-3# scratch along the scratch distance measured by the transducer, X-CT image analysis and probe profilometry.

Based on Equation (2), the fracture toughness values of the WCP specimens were calculated using the LEFM model, and the morphological parameters were obtained from both the empirical formula and X-CT image analysis, as shown in Figure 13. The corrected *K*_c_ and the statistical parameters are summarized in Table 4. The trend of the corrected *K*_c_ was consistent with the results of fitting with the empirical formula, demonstrating the feasibility of correcting the scratch groove morphology using X-CT image analysis and test results. The mean value of *K*_c_ values determined by empirical formula fitting was 0.30 MPa·m^1/2^. The corrected mean value was substantially lower, about 0.21 MPa·m^1/2^. While the mean value of 0.21 MPa·m^1/2^ obtained in this study aligns with the lower bound of the range (0.3–0.67 MPa·m^1/2^) reported for OPC in the literature [59,60,61,62,63] using notched three-point bending tests and dynamic molecular simulations, this discrepancy is mechanistically justifiable. Firstly, WCP typically exhibits a higher gypsum content and a distinct clinker phase composition compared to OPC, leading to a less tough microstructure. Secondly, and more critically, previous estimations often relied on empirical geometric relationships assuming no elastic recovery. X-CT analysis revealed an average elastic recovery rate of 26%, which significantly reduces the effective groove depth. Since effective groove depth is inversely proportional to the projected contact area, neglecting this recovery leads to a systematic overestimation. Therefore, the values reported here, corrected for both elastic recovery via X-CT, represent a more accurate intrinsic fracture property of WCP. The convergence of our results with the literature confirms that the X-CT methodology accurately captures the intrinsic fracture resistance of the material, offering a robust alternative for specimens where standard geometries are unattainable.

For the triangular groove profiles observed in the X-CT data, the groove cross-sectional area scaled quadratically with the groove width (*A*_LB_∝w^2^). According to Equation (2), *K*_c_ is inversely proportional to the square root of *A*_LB_. Consequently, *K*_c_ is inversely proportional to *w*. A relative error in width (Δ*w*/*w*) translates to an equal-magnitude relative error in *K*_c_, but in the opposite direction. The deviation in *K*_c_ corresponding to the width errors was calculated, as shown in Table 5. According to the sensitivity analysis above, a 10–20% overestimation in width directly results in an 18–36% overestimation of *K*_c_. This analysis definitively proves why correct geometry is paramount, which is critical for fracture mechanics assessments.

### 3.4. Probe Profilometer Analysis

**Figure 14:**
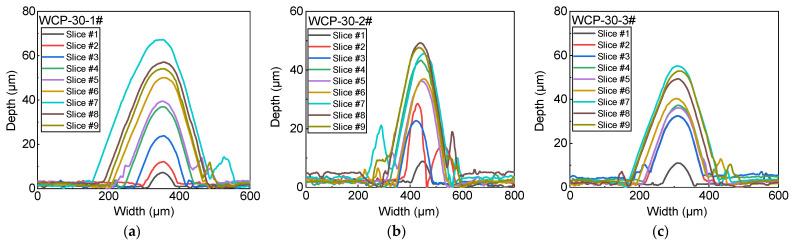
Morphological parameters of the grooves on ten sections of (**a**) WCP-30-1#, (**b**) WCP-30-2# and (**c**) WCP-30-3# scratch measured by a probe-type profilometer. Each section was at different distances from the starting point.

The displacement data acquired through the probe profilometer were converted into morphology data representing the scratch groove, as shown in Figure 14. The groove profiles obtained through probe profilometry and those analyzed via X-CT exhibited similar conical shapes, similar to the actual geometry of the probe. This similarity proved that the groove shapes obtained by X-CT image analysis were consistent with the actual results. The shapes of the grooves under different loads were generally similar. Some morphological changes may occur in damaged regions. Especially under high loads, a large amount of large debris fell off at the boundary of the grooves.

The width and depth parameters of the scratch grooves can be determined through probe profilometry. The measured depth values were compared with the data obtained from X-CT image analysis and the transducer during the scratch test, as shown in Figure 9. The variation trends of the depth measured by the three methods were consistent, and the depth gradually increased with increasing applied load. Under a load of 30 N, the maximum depth was above 80 μm across all methods. Owing to the nonfixed selection position of the probe profilometer, this study temporarily assumed that the cross-sections of the WCP were selected at equal intervals along the scratch length for the convenience of comparing the depth results. At low load levels, the depth results of the three methods showed little difference. However, as the load increased, the results from X-CT image analysis and the probe profilometer became essentially consistent, while the discrepancy with sensor measurements gradually increased. This phenomenon still indicates that WCP exhibits certain creep recovery characteristics. The main source of error is primarily due to the selection of cross-sectional positions.

**Figure 15:**
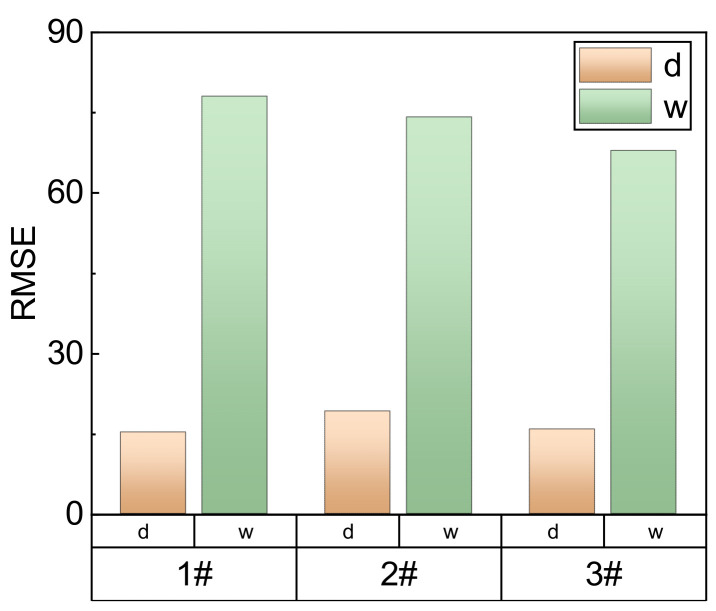
RMSE between the profilometer measurements by comparing against X-CT as the reference. d is the depth and w is the width.

The width of the scratch groove measured using the probe profilometer, X-CT and an empirical fitting formula were compared, as shown in Figure 11. The probe profilometer results were considerably greater than those derived from the empirical formula, which is consistent with the findings from the X-CT image analysis. Furthermore, as the probe profilometer captures surface topography through direct contact, its results are inferred to more accurately reflect the actual surface conditions, consistent with the larger width measurements obtained. Due to edge material delamination, the probe profilometer measured a noticeable abrupt change in groove width at certain delaminated locations. The experimental error of the profilometer measurements was evaluated by comparing against X-CT as the reference. The Root Mean Square Error (RMSE) for groove width and depth are shown in Figure 15. The primary sources of discrepancy include: the non-uniform sampling positions of the profilometer versus the evenly spaced X-CT slices and minor elastic rebound occurring between the scratch test and the profilometer scan. Given the above, the deviation between X-CT and profilometer is considered acceptable for microscratch morphological characterization of cementitious materials.

The shape parameter 2*pA*_LB_ measured using the probe profilometer was compared with the results derived from X-CT image analysis and empirical formula fitting, as shown in Figure 12. The morphological parameters of the groove obtained by the three measurement methods showed consistent trends along the scratch direction as the load increased. At low load levels during scratching, the groove morphology parameters were nearly identical. However, as the load increased, the differences among results from different analysis methods gradually widened, thereby affecting the calculated *K*_c_ for WCP. When depth parameters were all taken as instantaneous penetration depth, the results from the X-CT image analysis and probe profilometer were significantly higher than those derived from empirical formulas, due to the increased groove width measured by X-CT and profilometry. Due to the elastic recovery of CBMs, the depths obtained from X-CT and probe profilometer were noticeably smaller than the instantaneous penetration depth, leading to significantly reduced calculated groove morphology parameters—sometimes even lower than those predicted by the fitted empirical formula.

Using Equation (2), the *K*_c_ of the WCP was calculated. The results from the empirical formula, X-CT, and profilometric measurements were compared (Figure 13) and the trends were found to be essentially consistent, demonstrating the applicability of using X-CT image analysis to measure scratch groove morphology in CBMs with different compositions. The *K*_c_ obtained from the probe profilometer was similar to that derived from X-CT. The calculation of morphological parameters and fracture properties indicated that the results obtained from X-CT image analysis are consistent with profilometer measurements, suggesting the feasibility of X-CT image analysis for characterizing scratch groove morphology under the experimental conditions of this study. The advantage of X-CT lies in its 3D non-destructive imaging capability. The measurement result of a probe profilometer is height data on a line or a surface, and the selection of measurement positions cannot be precisely located as they are rather random. The probe may interact with the surface, posing a risk of scratching samples or wearing down the tip. X-CT can directly obtain the 3D spatial distribution of the overall material structure without any pretreatment, including the shape of pores.

### 3.5. Benchmarking Against Recent Advances

Recent studies have witnessed rapid evolution in both scratch-based and X-CT-based fracture characterization of cementitious materials. In scratch testing, Němeček et al. advanced phase-resolved microscratch characterization by coupling calibrated scratch tests with simultaneous acoustic emission recording, quantifying for individual cement paste constituents [24]. Wei et al. extended nanoscratch techniques to ITZ thickness quantification via a fracture toughness-based method [49]. These studies represent the state of the art in extracting phase-specific fracture properties from scratch tests, yet both rely on empirically assumed groove geometries without X-CT-based 3D correction. This is the limitation this work addresses. Our X-CT-corrected bulk values establish a validated baseline for interpreting phase-resolved data.

In X-CT-based fracture characterization, Yang et al. established the paradigm of in situ micro-X-CT for FPZ visualization, performing wedge split tests on UHPFRC with 16.9 μm voxel resolution and resolving the evolution from microcracks to macrocracks [64]. Deresse et al. pioneered the fusion of 4D-X-CT with acoustic emission and digital volume correlation, enabling simultaneous monitoring of fracture mode development during Brazilian splitting of cementitious mortars [65]. Hao et al. pushed the boundaries of X-CT image analysis by proposing a Transformer-based super-resolution model coupled with semantic segmentation, achieving automated, high-precision 3D crack recognition in ECC [66].

While these X-CT advances excel at visualizing fracture processes at macro scales or in fiber-reinforced composites, none have bridged X-CT-derived 3D morphology to microscratch-based LEFM parameter evaluation. This work adopts the X-CT segmentation philosophy and the in situ X-CT monitoring paradigm, then redirects them to the microscratch domain. Crucially, we validate the X-CT-derived geometries against stylus profilometry.

## 4. Conclusions

This study systematically integrates X-CT 3D reconstruction with microscratch testing and stylus profilometry validation to address the limitation of empirical geometric assumptions in cementitious material fracture toughness. The main conclusions are summarized as follows:

- The scratch response of WCP exhibited a clear dependence on the applied load. The penetration depth increased approximately linearly with scratch distance under linearly increasing loading conditions, while the lateral force showed a nonlinear power-law relationship with the normalized penetration depth (*d*/*R*). It indicates that the heterogeneous nature of WCP and the significant influence of material composition on scratch behavior.
- X-CT successfully captures 3D scratch groove morphology, overcoming the limitations of empirical geometric models. Relative to instantaneous sensor measurements, X-CT yields an average elastic recovery rate of 26.05% (attributed to reversible C-S-H deformation, partial microcrack closure, and stress redistribution within the cementitious matrix) and 15–20% larger groove widths (dominated by edge spalling and debris accumulation). These discrepancies amplify with increasing load, explaining the systematic *K*_c_ overestimation prevalent in prior microscratch studies.
- The mean X-CT-corrected *K*_c_ was 0.21 MPa·m^1/2^, about 30% lower than empirical fitting-derived values, representing a more accurate intrinsic fracture property of WCP. Sensitivity analysis confirms geometric errors dominate uncertainty. A 10% overestimation in groove width induces an 18% overestimation in *K*_c_ validating the critical role of 3D geometric correction for fracture mechanics assessment.
- The groove morphology and fracture parameters obtained from X-CT showed good agreement with those measured by probe profilometry. This agreement validates the reliability of the X-CT-based approach and confirms its capability to accurately characterize scratch-induced damage in cementitious materials.

Overall, the proposed X-CT-assisted scratch test methodology provides a reliable framework for evaluating the fracture behavior of cementitious materials and has significant potential for application in multi-scale characterization and durability assessment of advanced cement-based composites.

The X-CT-assisted scratch testing methodology proposed herein transcends the limitations of conventional macro-scale fracture tests by enabling micro-destructive, high-fidelity characterization of cementitious materials. Its primary engineering value lies in the ability to extract intrinsic fracture toughness from miniature samples, facilitating the assessment of aging infrastructure where large-scale sampling is prohibited. While the current reliance on benchtop micro-CT and manual segmentation limits its throughput, rendering it primarily a research tool for micro-mechanical investigation, ongoing advancements in portable CT technology and AI-driven image analysis promise significant improvements. Future integration with standardized scratch testing protocols could establish this technique as a routine inspection tool, bridging the critical gap between nano-mechanical probing and structural health monitoring.

A limitation of this study is the incomplete nature of complementary microstructural characterization. While SEM confirmed the uniform distribution of unhydrated cement particles in the matrix and microcracking, the absence of energy-dispersive X-ray spectroscopy (EDS) prevents quantitative correlation of micro-scale elemental segregation with localized fluctuations across parallel scratches. The lack of X-ray diffraction (XRD) data further limits our ability to evaluate the influence of trace crystalline phases on scratch response. Future work will integrate EDS microanalysis, XRD quantitative phase identification, focused ion beam-SEM (FIB-SEM) for site-specific cross-sectioning, and synchrotron-based X-CT to further refine mechanistic understanding.

It is important to acknowledge the specific boundary conditions under which this study was conducted. The experimental program was restricted to a single WCP formulation with a w/c of 0.4, tested at 28 days of curing using a Rockwell sphero-conical indenter under a fixed loading rate. Consequently, the quantitative values for elastic recovery and the absolute *K*_c_ values are specific to this matrix and testing configuration. Variations in w/c ratio would alter the pore structure and stiffness, potentially affecting the magnitude of recovery and spalling, although the fundamental mechanisms are expected to be universal for cementitious materials. Future research should extend this X-CT-assisted scratch testing framework to composite systems (mortars and concretes), and investigate the influence of early-age hydration and long-term curing, to establish a comprehensive understanding of micro-scale fracture across various scales and material compositions.

## Figures and Tables

**Table 1:** Physical properties of WCP.

Fineness (0.08/%)	Density (g/cm^3^)	Specific Surface Area (m^2^/kg)	Flexural Strength (MPa)		Compressive Strength (MPa)	
			3 d	28 d	3 d	28 d
0.42	3.02	420	4.8	7.2	0.42	3.02

**Table 2:** Chemical composition and mineral of WCP (wt.%).

SiO_2_	Al_2_O_3_	Fe_2_O_3_	CaO	MgO	SO_3_	Na_2_Oeq	f-CaO	C_3_S	C_2_S	C_3_A	C_4_AF
15.96	1.84	0.27	72.17	3.91	3.84	0.27	0.96	58.77	23.57	12.03	0.5

**Table 3:** Fitting parameters for the displacement–scratch-distance curve with statistical parameters.

**Scratch Number**	**LFE** **1**								
	**k**	**Mean**	**SD**	**95%CI**	**m**	**Mean**	**SD**	**95%CI**	**R^2^**
WCP-30-1#	54.47 ± 0.23	58.90	4.10	[48.82, 68.98]	0	0	0	None	0.9942
WCP-30-2#	59.79 ± 0.33				0				0.9869
WCP-30-3#	62.44 ± 0.30				0				0.9951
**Scratch Number**	**LFE** **2**								
	**k**	**Mean**	**SD**	**95%CI**	**m**	**Mean**	**SD**	**95%CI**	**R^2^**
WCP-30-1#	22.41 ± 0.49	24.29	2.10	[19.07, 29.51]	48.08 ± 1.16	46.17	5.04	[33.65, 58.69]	0.8563
WCP-30-2#	23.91 ± 0.41				49.98 ± 0.94				0.8896
WCP-30-3#	26.56 ± 0.22				40.45 ± 0.52				0.9805

Note: LFE—linear fitting equation; k—slope; m—intercept; SD—standard deviation of three specimens; 95%CI—95% confidence interval.

**Table 4:** *K*_c_ and the statistical parameters from scratch tests and X-CT analysis.

Scratch Number	*K*_c_ (MPa⋅m^1/2^)	Mean (MPa⋅m^1/2^)	SD (MPa⋅m^1/2^)	95%CI	*K*_c,XCT_ (MPa⋅m^1/2^)	Mean (MPa⋅m^1/2^)	SD (MPa⋅m^1/2^)	95%CI
WCP-30-1#	0.31 ± 0.11	0.30	0.12	[0.21, 0.39]	0.21 ± 0.017	0.21	0.017	[0.19, 0.23]
WCP-30-2#	0.28 ± 0.07				0.20 ± 0.017			
WCP-30-3#	0.30 ± 0.15				0.21 ± 0.016			

**Table 5:** Sensitivity of calculated fracture toughness to geometric measurement errors in groove width.

Relative Error in Measured Width (Δ*w*/*w*)	Relative Error in Calculated (Δ*K*_c_/*K*_c_)	Absolute Deviation for WCP (Baseline *K*_c_ = 0.25 MPa·m^1/2^)
+5%	−9.5%	−0.024 MPa·m
+10%	−18.2%	−0.045 MPa·m
+20%	−35.6%	−0.089 MPa·m

## Data Availability

The original contributions presented in this study are included in the article. Further inquiries can be directed to the corresponding author.
